# Functionalized Aluminum Nitride for Improving Hydrolysis Resistances of Highly Thermally Conductive Polysiloxane Composites

**DOI:** 10.1007/s40820-025-01669-5

**Published:** 2025-02-06

**Authors:** Mukun He, Lei Zhang, Kunpeng Ruan, Junliang Zhang, Haitian Zhang, Peng Lv, Yongqiang Guo, Xuetao Shi, Hua Guo, Jie Kong, Junwei Gu

**Affiliations:** 1 Shaanxi Key Laboratory of Macromolecular Science and Technology, School of Chemistry and Chemical Engineering, Northwestern Polytechnical University, Xi’an, 710072 Shaanxi People’s Republic of China; 2 Chongqing Innovation Center, Northwestern Polytechnical University, Chongqing, 401135 People’s Republic of China; 3 School of Materials Science and Engineering, Shandong University of Technology, Zibo, 255000 People’s Republic of China

**Keywords:** Polymethylhydrosiloxane, Aluminum nitride, Copolymer, Thermally conductive composites

## Abstract

A series of divinylphenyl-acryloyl chloride copolymers (PDVB-*co*-PACl) is synthesized *via* atom transfer radical polymerization employing tert-butyl acrylate and divinylbenzene as monomers. PDVB-*co*-PACl is utilized to graft on the surface of spherical aluminum nitride (AlN) to prepare functionalized AlN (AlN@PDVB-*co*-PACl). Polymethylhydrosiloxane (PMHS) is then used as the matrix to prepare thermally conductive AlN@PDVB-*co*-PACl/PMHS composites with AlN@PDVB-*co*-PACl as fillers through blending and curing. The grafting of PDVB-*co*-PACl synchronously enhances the hydrolysis resistance of AlN and its interfacial compatibility with PMHS matrix. When the molecular weight of PDVB-*co*-PACl is 5100 g mol^−1^ and the grafting density is 0.8 wt%, the composites containing 75 wt% of AlN@PDVB-*co*-PACl exhibit the optimal comprehensive performance. The thermal conductivity (*λ*) of the composite is 1.14 W m^−1^ K^−1^, which enhances by 20% and 420% compared to the *λ* of simply physically blended AlN/PMHS composite and pure PMHS, respectively. Meanwhile, AlN@PDVB-*co*-PACl/PMHS composites display remarkable hydrothermal aging resistance by retaining 99.1% of its *λ* after soaking in 90 °C deionized water for 80 h, whereas the *λ* of the blended AlN/PMHS composites decreases sharply to 93.7%.

**Supplementary Information:**

The online version contains supplementary material available at 10.1007/s40820-025-01669-5.

## Highlights

- Copolymer of divinylphenyl-acryloyl chloride copolymers (PDVB-*co*-PACl) is designed and synthesized to graft on the surface of aluminum nitride (AlN) to improve its hydrolysis resistance.
- AlN fillers functionalized by PDVB-*co*-PACl with the molecular weight of 5100 g mol^-1^ exhibits the highest hydrolysis resistance and the lowest interfacial thermal resistance.
- When the mass fraction of AlN@PDVB-*co*-PACl is 75 wt% and the grafting density of PDVB-*co*-PACl is 0.8 wt%, the *λ* for AlN@PDVB-*co*-PACl/PMHS composites is 1.14 W m^-1^ K^-1^ and maintains 99.1% after soaking in 90 °C deionized water for 80 h.

**Supplementary Information:**

The online version contains supplementary material available at 10.1007/s40820-025-01669-5.

## Graphical abstract

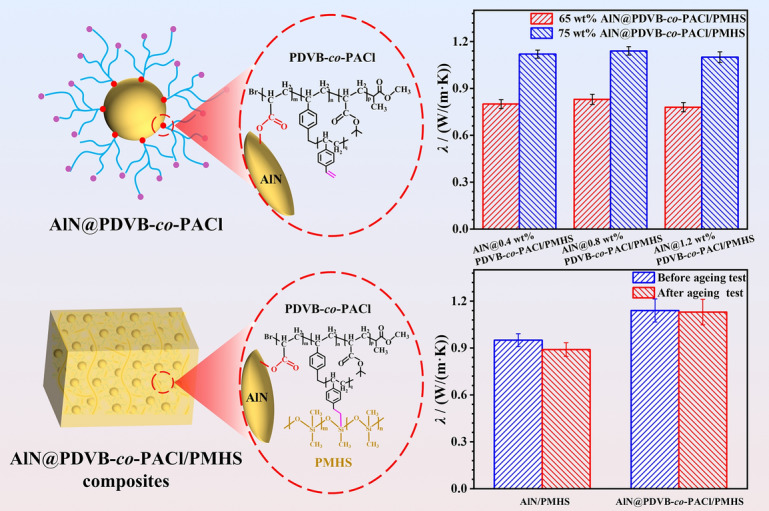

## Introduction

With the rapid development of electronic products toward high power and integration, the problems of heat accumulation and heat dissipation become more and more prominent [1–5], which puts forward higher requirements on the thermal conductivity, insulation, and heat resistance of the thermal interface materials [6–10]. Compared with traditional thermal interface materials (such as epoxy resin, polyurethane, etc. [11]), polymethylhydrosiloxane (PMHS) is an ideal matrix for the preparation of thermal interface materials because of its excellent insulating property, aging resistance, ease of molding, and compatibility with silicon substrates [12–14]. However, it is usually necessary to fill a large number of thermally conductive fillers into PMHS to obtain high thermal conductivity [15–17]. Aluminum nitride (AlN) as a material with high thermal conductivity (*λ*, 170 W m^−1^ K^−1^) and excellent electrical insulation, is an ideal filler for the preparation of PMHS composites with high thermal conductivity to be used as thermally conductive polysiloxane sheets, which play an effective role in heat dissipation to ensure the normal operation of the equipment [18–20]. However, the poor compatibility of AlN with PMHS is not beneficial to heat transfer, which limits the efficient improvement of thermal conductivity for AlN/PMHS composites [21, 22]. In addition, AlN is prone to hydrolysis in humid air, thus reducing its thermal conductivity and affects the stability of electronic devices after a long period of time with a rapid decline in performance [23–25].

The existing methods to modify the surface of AlN mainly involve physical coating and chemical grafting [26–28]. Physical coating is to combine functional substances on AlN surface by physical interaction [29–31]. Ganesh et al. [32] treated AlN powder by simultaneously using phosphoric acid and aluminum dihydrogen phosphate in ethanol solution. The treated AlN powder was found to remain stable after 72 h in water. However, physical coating suffers from low binding of the surface cladding layer to AlN and poor modification effect [33–35]. Chemical grafting is to graft small molecules or polymers onto the surface of AlN through chemical reaction with the modified layer firmly bound to AlN [36–38]. Lin et al. [39] prepared AlN-VTMS/PMHS composites using PMHS as matrix and vinyl trimethoxy-silane (VTMS) modified AlN (AlN-VTMS) as thermally conductive fillers. When the mass fraction of AlN-VTMS was 60 wt%, the thermal conductivity (*λ*) of AlN-VTMS/PMHS composites was 0.31 W m^−1^ K^−1^, which was higher than 0.24 W m^−1^ K^−1^ for unmodified AlN/PMHS composites with the same filler amount. Yang et al. [40] prepared AlN@(PEDOT:PSS)/PU composites using polyurethane (PU) as matrix and poly(3,4-vinyldioxthiophene):polystyrene sulfonic acid (PEDOT:PSS) modified AlN (AlN@(PEDOT:PSS)) as thermally conductive fillers. When the grafting density of PEDOT:PSS was 0.5 wt% and the mass fraction of fillers was 50 wt%, the *λ* of AlN@(PEDOT:PSS)/PU composites was 0.76 W m^−1^ K^−1^, which was higher than 0.48 W m^−1^ K^−1^ for the unmodified AlN/PU composites applying the same amount of fillers. However, the existing chemical grafting methods cannot simultaneously improve the hydrolysis resistance of AlN and its interfacial compatibility with PMHS matrix [41].

Copolymer contains abundant chemically reactive terminal groups [42–44], which can significantly improve the interfacial compatibility with the polymer matrix when grafted on the surface of thermally conductive fillers [45–47]. Meanwhile, copolymer polymers with branched structures have the advantages of small hydrodynamic radius and low viscosity, which provide excellent processing properties [48–50]. Hu et al. [51] prepared boron nitride fillers with the surface coated by copolymer of tea polyphenol and theophylline (BN@TPP) to make thermally conductive composites with polydimethylsiloxane as matrix (BN@TPP/PDMS). The *λ* of BN@TPP/PDMS composites with 40 wt% of BN@TPP increased to 0.46 from 0.35 W m^−1^ K^−1^ for the composite with the same amount of unmodified BN as fillers. Zhang et al. [52] applied 3-(Trimethoxysilyl)propyl methacrylate grafted poly(methylhydrosiloxane-co-dimethylsiloxane) (MPS-*g*-PHMS-PDMS) by hydrosilylation reaction. Further, Al_2_O_3_@MPS-*g*-PMHS-PDMS composites were prepared by blending and curing with SR as matrix and MPS-*g*-PMHS-PDMS modified Al_2_O_3_ (Al_2_O_3_@MPS-*g*-PMHS-PDMS) as thermally conductive fillers. When the mass fraction of Al_2_O_3_ was 83 wt% and the grafting density of MPS-*g*-PMHS-PDMS was 2.5 wt%, the Al_2_O_3_@MPS-*g*-PMHS-PDMS/SR composites exhibited an increased *λ* of 1.68 W m^−1^ K^−1^ and a decreased viscosity of 73.1 Pa s from 1.42 W m^−1^ K^−1^ to 85.0 Pa s for the Al_2_O_3_/SR composites with the same amount of unmodified Al_2_O_3_, respectively.

Herein, a series of copolymers with different molecular weight were synthesized by atom transfer radical polymerization (ATRP) using tert-butyl acrylate (*t*BA) and divinylbenzene (DVB) as monomers, followed by acylation of the tert-butyl group to afford PDVB-*co*-PACl. PDVB-*co*-PACl was then employed to graft on the surface of AlN to prepare (AlN@PDVB-*co*-PACl). Further, thermally conductive AlN@PDVB-*co*-PACl/PMHS composites were prepared with PMHS as matrix by blending and curing. PDVB-*co*-PACl was characterized and analyzed by nuclear magnetic resonance (NMR), Fourier transform infrared (FT-IR) spectroscopy and size exclusion chromatography (SEC). The surface structure and morphology of AlN@PDVB-*co*-PACl were characterized by X-ray diffraction (XRD), X-ray photoelectron spectroscopy (XPS) and transmission electron microscopy (TEM). Furthermore, the effects of molecular weight, grafting density, and mass fraction of AlN@PDVB-*co*-PACl on the thermal conductivity, viscosity, mechanical properties, and thermal properties of AlN@PDVB-*co*-PACl/PMHS composites were analyzed and investigated, and the thermal conductivity mechanism was analyzed simultaneously.

## Experimental Section

### Synthesis of PDVB-co-PACl

In argon atmosphere, tert-butyl acrylate (*t*BA, 35.75 mmol), cupric bromide (CuBr, 1.26 mmol), pentamethyldivinyltriamine (PMDETA, 0.81 mmol), methyl 2-bromopropionate (3.31 mmol), divinylbenzene (DVB, 3.31 mmol), and N,N-dimethylacetamide (DMAc, 22.99 mmol) were introduced to a Schlenk bottle and stirred evenly. The mixture reacted at 60 °C for 5 h and then poured into a large amount of deionized water to precipitate the polymer. The precipitated polymer was filtered, then dissolved in tetrahydrofuran (THF), and poured into deionized water again to precipitate the polymer, and repeated four times; then dried under vacuum to obtain PDVB-*co*-P*t*BA. Then, PDVB-*co*-P*t*BA (0.41 mmol), THF (12.47 mmol), and thionyl chloride (SOCl_2_, 0.031 mmol) were added into a single-necked flask with stirred and sealed, and then the reaction was carried out at room temperature for 2 h before THF was removed by rotary evaporation. The solid residue was dissolved with appropriate amount of THF, which was removed using rotary evaporation to afford PDVB-*co*-PACl with molecular weight of 2800, 5100, and 7900 g mol^−1^ realized by adjusting the amount of initiator, recorded as PDVB-*co*-PACl-1, PDVB-*co*-PACl-2, and PDVB-*co*-PACl-3, respectively.

### Preparation of AlN@PDVB-co-PACl

**Fig. 1:**
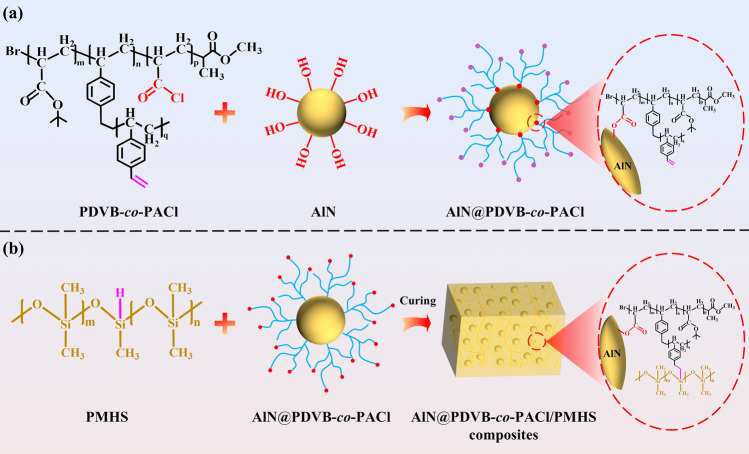
Schemes for the preparation of **a** AlN@PDVB-*co*-PACl fillers and **b** AlN@PDVB-*co*-PACl/PMHS composites

PDVB-*co*-PACl (0.031 mmol), THF (0.28 mmol), and AlN (0.61 mmol) were added into a flask and stirred evenly. The reaction was carried out at 50 °C for 2 h. The functionally-modified AlN (AlN@PDVB-*co*-PACl) was obtained by filtration, washing, and vacuum drying. The preparation process is shown in Fig. 1a. AlN@0.4 wt% PDVB-*co*-PACl, AlN@0.8 wt% PDVB-*co*-PACl and AlN@1.2 wt% PDVB-*co*-PACl were prepared by adjusting the grafting density on AlN surface.

### Fabrication of AlN@PDVB-co-PACl/PMHS Composites

The PMHS matrix and AlN@PDVB-*co*-PACl fillers were added into a beaker according to the predetermined proportion and stirred evenly, then poured into a polytetrafluoroethylene mold. After vacuum defoaming, the thermally conductive AlN@PDVB-*co*-PACl/PMHS composites were fabricated by heat curing at 120 °C for 2 h, as shown in Fig. 1b.

## Results and Discussion

### Characterization of PDVB-co-PACl

**Fig. 2:**
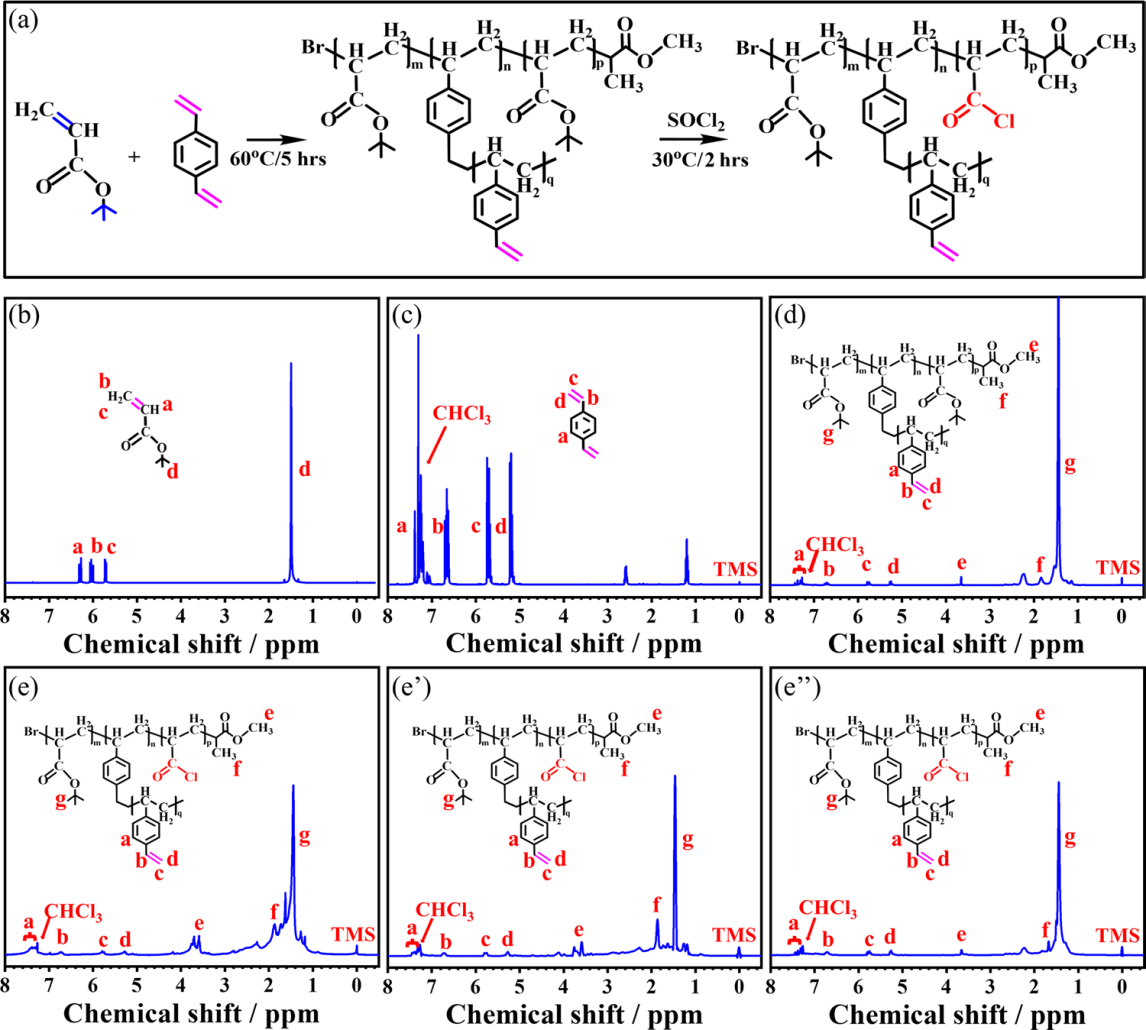
**a** Schematic diagram of synthetic route of PDVB-*co*-PACl. ^1^H NMR spectra of **b** *t*BA, **c** DVB, **d** PDVB-*co*-P*t*BA, **e** PDVB-*co*-PACl with *M*_n_ of 2800, **e’** PDVB-*co*-PACl with *M*_n_ of 5100 and **e’’** PDVB-*co*-PACl with *M*_n_ of 7900

Figure 2a shows a schematic diagram of the synthetic route for PDVB-*co*-PACl by ATRP using tert-butyl acrylate (*t*BA) and divinylbenzene (DVB) as monomers, meanwhile methyl 2-bromopropionate as initiators. The first step is a copolymerization reaction to prepare PDVB-*co*-P*t*BA. The second step is a chlorination reaction, which is prepared by chlorinating PDVB-*co*-P*t*BA using the chlorination reagent sulfoxide chloride to obtain PDVB-*co*-PACl. The sum of m and p is the number of moles for *t*BA divided by the number of moles for initiators. The sum of n and q is the number of moles for DVB divided by the number of moles for initiators. Figure 2b–e**’’** display the ^1^H NMR spectra of *t*BA, DVB, PDVB-*co*-P*t*BA, and PDVB-*co*-PACl with different molecular weights. In Fig. 2b, the peaks at 6.38, 6.09, and 5.71 ppm correspond to the protons on the vinyl group of *t*BA. The peak at 1.48 ppm corresponds to the proton in the tert-butyl group of *t*BA. In Fig. 2c, the peaks at 7.38 and 6.54 ppm correspond to protons on the benzene ring of DVB, while the peaks at 5.72 and 5.28 ppm correspond to prbbotons on the ethenyl group of DVB. After copolymerization, in Fig. 2d, the peaks at 7.45 and 7.12 ppm correspond to protons on the benzene ring of DVB, while the peaks at 5.68 and 5.14 ppm correspond to protons on the vinyl group of DVB. Additionally, the peak at 1.48 ppm corresponds to the proton on the tert-butyl group of the *t*BA. Meanwhile, the integrations of the peaks of (a + b):(c + d):e:f:g are 3.88:1.00:0.97:0.94:94.32, which indicates the successful synthesis of PDVB-*co*-P*t*BA. The integration of the peaks of (a + b):(c + d):e:f:g are 3.81:1.00:1.09:0.55:12.56 (Fig. 2e), 3.85:1.00:0.95:0.43:13.78 (Fig. 2e**’**), 3.87:1.00:0.88:0.23:14.23 (Fig. 2e**’’**) for PDVB-*co*-PACl-1, PDVB-*co*-PACl-2 and PDVB-*co*-PACl-3, respectively. According to the integration difference of the tert-butyl group before and after acylation, the acylation degree for PDVB-*co*-PACl-1, PDVB-*co*-PACl-2, and PDVB-*co*-PACl-3 are found to be 81%, 79%, and 75%.

Size exclusion chromatography (SEC) is further employed to characterize the molecular weight and molecular weight distribution of PDVB-*co*-PACl (Fig. S1). The *M*_n_ of PDVB-*co*-PACl-1, PDVB-*co*-PACl-2, and PDVB-*co*-PACl-3 are 2800, 5100, and 7900 g mol^−1^, respectively. The SEC curves display relatively wide polydispersity (*Ð*) of 2.04, 1.94, and 2.17, respectively, which is mainly due to the fact that PDVB-*co*-PACl contains a large number of side chains. Figure S2 shows the FT-IR spectra of *t*BA, DVB, PDVB-*co*-P*t*BA and PDVB-*co*-PACl. After ATRP polymerization, the characteristic peak of tert-butyl group in PDVB-*co*-P*t*BA appears at 2960 cm^−1^, and the characteristic peak of carbonyl in *t*BA appears at 1736 cm^−1^. And the characteristic peaks of benzene ring skeleton vibration in DVB appear at 1600, 1580, 1500, and 1450 cm^−1^, indicating the successful synthesis of PDVB-*co*-P*t*BA. More importantly, the characteristic peak of carbon group in the chlorination group for PDVB-*co*-PACl appears at 1755 cm^−1^, which in combination with ^1^H NMR spectra and SEC spectra indicate the successful conduct of the chlorination reaction and the successful synthesis of PDVB-*co*-PACl.

### Characterization and Hydrolysis Resistance of AlN@PDVB-co-PACl

**Fig. 3:**
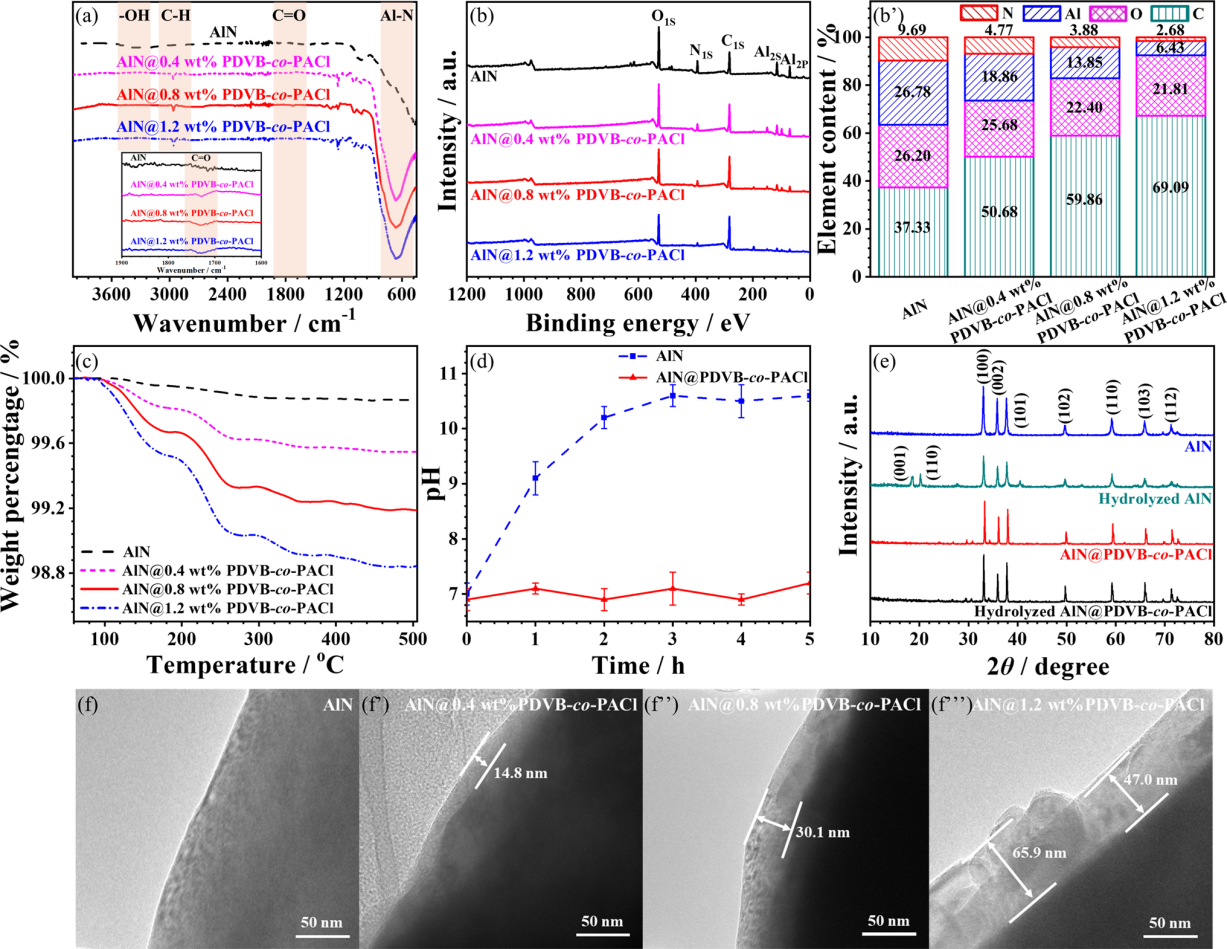
**a** FT-IR spectra, **b** XPS spectra, **b’** elements concentration and **c** TGA curves of AlN and AlN@PDVB-*co*-PACl. **d** pH and **e** XRD spectra of AlN and AlN@PDVB-*co*-PACl before and after hydrolysis. TEM images of **f** AlN and **f’-f’’’** AlN@PDVB-*co*-PACl with different grafting density

FT-IR, XPS, TGA are applied to investigate the structure and physical properties of AlN@PDVB-*co*-PACl. Figure 3a shows the FT-IR spectra of AlN and AlN@PDVB-*co*-PACl. Both AlN and AlN@PDVB-*co*-PACl show strong characteristic absorption peak at 670 cm^−1^, corresponding to the stretching vibration of the AlN bond. Compared with AlN, AlN@PDVB-*co*-PACl shows a characteristic absorption peak of the carbonyl group at 1730 cm^−1^, corresponding to the carbonyl group in PDVB-*co*-PACl, indicating that PDVB-*co*-PACl is successfully grafted on the surface of AlN. At the same time, the characteristic absorption peak of AlN@PDVB-*co*-PACl at 670 cm^−1^ for the AlN bond broadens and the hydroxyl peak decreases. This is mainly attributed to the formation of covalent bonds by condensation reactions between the hydroxyl groups on the AlN surface and the chloride groups [53]. Figure 3b displays the XPS spectra of AlN and AlN@PDVB-*co*-PACl, the corresponding element content is shown in Fig. 3b**’**, and the corresponding O 1*s* spectrum is shown in Fig. S3. Compared with AlN, the content of C for AlN@PDVB-*co*-PACl increases, while the content of Al, O, and N decreases, mainly due to the higher C content of PDVB-*co*-PACl on AlN@PDVB-*co*-PACl surface. In addition, the O 1*s* characteristic peaks of AlN (Fig. S3a) appearing at 532.2 and 531.6 eV corresponds to Al-O–H and Al-O-N bonds, respectively. After the grafting of PDVB-*co*-PACl, the O 1*s* characteristic peaks of AlN@PDVB-*co*-PACl (Fig. S3b) appearing at 533.1, 531.8, and 531.3 eV correspond to the C = O, C-O, and Al-O-N bonds, respectively. The appearance of the C = O bond and the disappearance of the Al–O–H bond on the AlN surface indicate that the condensation reaction between the acyl chloride groups of PDVB-*co*-PACl and the hydroxyl groups successfully grafted on the AlN surface.

As shown in Fig. 3c, there is only a slight mass loss of AlN before 350 °C and essentially no mass loss after 350 °C showing 99.9 wt% residual, which is mainly attributed to the evaporation of adsorbed water molecules on the AlN surface and the loss of hydroxyl groups. After grafting PDVB-*co*-PACl, the AlN@0.4 wt% PDVB-*co*-PACl, AlN@0.8 wt% PDVB-*co*-PACl and AlN@1.2 wt% PDVB-*co*-PACl exhibit weight loss at 100 ~ 460 °C. It is mainly attributed to the breakage of the covalent bond between AlN and PDVB-*co*-PACl and the cleavage of PDVB-*co*-PACl. However, no mass loss is observed for AlN@PDVB-*co*-PACl after 460 °C. In addition, AlN@PDVB-*co*-PACl presents 99.6, 99.2, and 98.8 wt% loss weight, suggesting the grafting density of PDVB-*co*-PACl are 0.4, 0.8, and 1.2 wt%, respectively. Figure 3d shows the pH values at different time for the mixture of AlN and AlN@PDVB-*co*-PACl in deionized water at 40 °C. As observed, the pH for AlN rise gradually and maintain at around 10.5 after 2 h. In contrast, the pH for AlN@PDVB-*co*-PACl barley changes and keeps constant between 6.8 and 7.2. In addition, PDVB-*co*-PACl prevents water molecules from penetrating into the internal AlN, which effectively prevents its hydrolysis. Figure 3e shows the XRD curves of AlN and AlN@PDVB-*co*-PACl before and after hydrolysis. The strong diffraction peaks at 33.3°, 36.1°, 37.8°, 49.8°, 59.8°, 66.0°, 69.8°, 71.7°, and 72.8° for AlN and AlN@PDVB-*co*-PACl correspond to the (100), (002), (101), (102), (110), (103), and (112) crystal planes, respectively. After hydrolysis, strong diffraction peaks at 18.6°, 21.8°, and 41.2° for AlN correspond to the (001), (110), and (201) crystal planes for Al(OH)_3_, respectively, indicating that AlN reacts with H_2_O. In contrast, no Al(OH)_3_ diffraction peaks in the XRD pattern of AlN@PDVB-*co*-PACl are observed after hydrolysis test, also indicating no obvious hydrolysis occurs for AlN@PDVB-*co*-PACl. This is ascribed to hydrophobic groups of benzene ring and abundant branched chain network in PDVB-*co*-PACl, which increase its hydrophobicity (Fig. S4). The above results demonstrate that the grafting of PDVB-*co*-PACl significantly increase the hydrolysis resistance of AlN. TEM is then applied to observe the surface morphology for AlN and AlN@PDVB-*co*-PACl (Fig. 3f–f**’’’**). As observed, the surface of AlN is smooth and free with other substances. By comparison, a grafting layer of PDVB-*co*-PACl is visible on the surface of AlN@PDVB-*co*-PACl, with the thickness increasing from 14.8 to 30.1 to 47.0 ~ 65.9 nm as the grafting density increases from 0.4 to 0.8 to 1.2 wt%. Because the thickness of the grafting layer becomes thicker with the increase in grafting density, but too much grafting can lead to agglomeration resulting in uneven thickness.

### Thermal Conductivity of AlN@PDVB-co-PACl/PMHS Composites

**Fig. 4:**
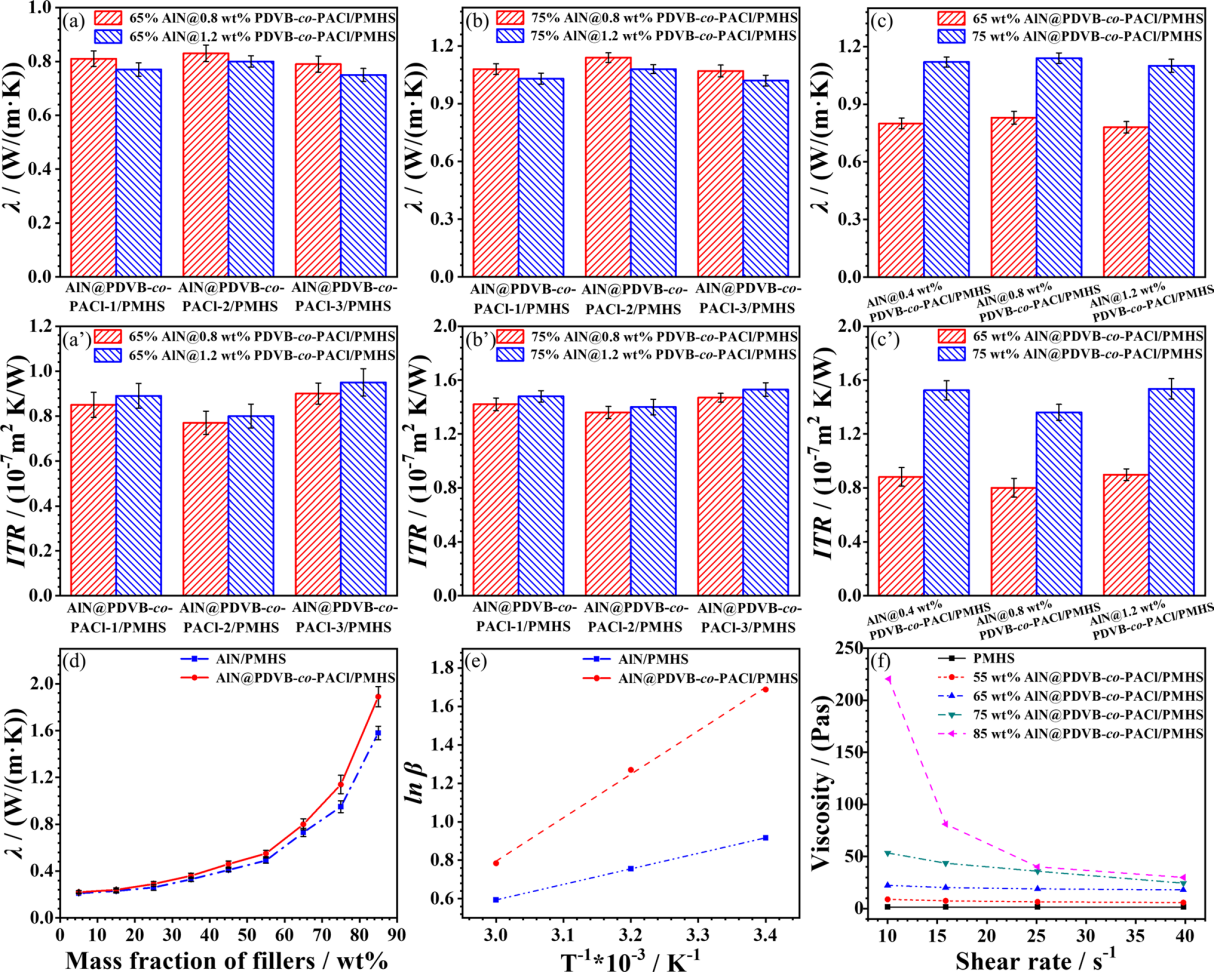
**a-b** *λ* and **a’-b’** *ITR* of AlN@PDVB-*co*-PACl/PMHS composites with different molecular mass PDVB-*co*-PACl. **c** *λ* and **c’** *ITR* of AlN@PDVB-*co*-PACl/PMHS composites with different grafting density of PDVB-*co*-PACl. **d** *λ* of AlN/PMHS and AlN@PDVB-*co*-PACl/PMHS composites. **e** Curves of logarithm of bound PMHS versus inverse temperature. **f** Curves of viscosity versus shear rate for AlN@PDVB-*co*-PACl/PMHS composites

The effects of different molecular weight and grafting density AlN@PDVB-*co*-PACl, as well as mass fraction AlN@PDVB-*co-*PACl on thermal conductivity (*λ*) and interfacial thermal resistance (*ITR*) are investigated. Figure 4a–b and 4a’–b’ shows the *λ* and *ITR* of AlN@PDVB-*co*-PACl/PMHS composites at different molecular weights of PDVB-*co*-PACl. Under the same grafting density of PDVB-*co*-PACl and the mass fraction of AlN@PDVB-*co*-PACl, the *λ* of AlN@PDVB-*co*-PACl/PMHS composite first increases and then decreases while the corresponding *ITR* first decreases and then increases with the increase in molecular weight of PDVB-*co*-PACl. When the molecular weight and grafting density of PDVB-*co*-PACl are 5100 g mol^−1^ and 0.8 wt% and the mass fraction of AlN@PDVB-*co*-PACl is 75 wt%, respectively, the *λ* of AlN@PDVB-*co*-PACl/PMHS composites reached the maximum of 1.14 W m^−1^ K^−1^, and the corresponding *ITR* is 1.36 × 10^–7^ m^2^ K W^−1^. Superior to the *λ* and *ITR* of AlN@PDVB-*co*-PACl/PMHS at the same mass fraction of AlN with PDVB-*co*-PACl molecular weight of 2800 g mol^−1^ (1.08 W m^−1^ K^−1^, 1.42 × 10^–7^ m^2^ K W^−1^) and PDVB-*co*-PACl molecular weight of 7900 g mol^−1^ (1.07 W m^−1^ K^−1^, 1.45 × 10^–7^ m^2^ K W^−1^). This is because PDVB-*co*-PACl-1 with the *M*_n_ of 2800 g mol^−1^ has fewer acyl chloride and vinyl functional groups, resulting in weak chemical bonding between AlN and PMHS matrix. Meanwhile, the relative shorter chains are difficult to form effective physical entanglements with the PMHS matrix. When the *M*_n_ of PDVB-*co*-PACl-2 is 5100 g mol^−1^, the length of main chains and branched chains of PDVB-*co*-PACl-2 is moderate, and it is easy to form physical entanglements with PMHS matrix. However, as the *M*_n_ increases further to 7900 g mol^−1^, the longer polymer chains make it difficult to diffuse within PMHS matrix to make AlN disperse well, which is not conducive to the efficient formation of AlN-AlN thermally conductive pathways. Therefore, PDVB-*co*-PACl-2 with the *M*_n_ of 5100 g mol^−1^ is selected for the following study. Figure 4c–c**’** shows the *λ* and *ITR* of AlN@PDVB-*co*-PACl/PMHS composites with different PDVB-*co*-PACl grafting density. The *λ* of AlN@PDVB-*co*-PACl/PMHS composites tend to increase and then decrease with the increase in PDVB-*co*-PACl grafting density, and the corresponding *ITR* tends to decrease and then increase. When the grafting density of PDVB-*co*-PACl is 0.8 wt% and the mass fraction is 75 wt%, the* λ* of AlN@0.8 wt% PDVB-*co*-PACl/PMHS composites reaches the maximum, which is 1.14 W m^−1^ K^−1^, and the corresponding *ITR* is 1.36 × 10^–7^ m^2^ K W^−1^. It is better than the *λ* and corresponding *ITR* (1.12 W m^−1^ K^−1^ and 1.40 × 10^–7^ ^2^ K W^−1^) of AlN@0.4 wt% PDVB-*co*-PACl/PMHS as well as *λ* and corresponding *ITR* (1.10 W m^−1^ K^−1^ and 1.43 × 10^–7^ m^2^ K W^−1^) of AlN@1.2 wt% PDVB-*co*-PACl/PMHS. When the grafting density of PDVB-*co*-PACl is 0.8 wt%, PDVB-*co*-PACl formed a complete and uniform thickness grafting layer on the AlN surface (Fig. 3f**’’**), which effectively improved the interfacial compatibility between AlN and PMHS matrix, reduced *ITR*, and conferred a higher *λ* to AlN@PDVB-*co*-PACl/PMHS composites. In contrast, the grafting layer of PDVB-*co*-PACl on the AlN surface is incomplete when the grafting density of PDVB-*co*-PACl is low (0.4 wt%, Fig. 3f**’**), and the thickness of the grafting layer of PDVB-*co*-PACl is not uniform when the grafting density is high (1.2 wt%, Fig. 3f**’’’**). All of them are unfavorable to the efficient interfacial bonding between AlN and PMHS matrix, and cannot improve the interfacial compatibility between AlN and PMHS matrix efficiently, and the *λ* of the corresponding PMHS composites is low. In summary, when the grafting density of PDVB-*co*-PACl is 0.8 wt%, AlN@PDVB-*co*-PACl/PMHS with the same amount of AlN has the best thermal conductivity, so 0.8 wt% is selected as the optimal grafting density.

Figure 4d shows *λ* of AlN/PMHS and AlN@PDVB-*co*-PACl/PMHS composites with different mass fraction. When the mass fraction of AlN@PDVB-*co*-PACl is 75 wt%, AlN@PDVB-*co*-PACl/PMHS composites have the best thermal conductivity, *λ* is 1.14 W m^−1^ K^−1^ and *ITR* is 1.36 × 10^–7^ m^2^ K W^−1^, which is better than that of *λ* and *ITR* for 75 wt% AlN/PMHS (0.95 W m^−1^ K^−1^ and 1.67 × 10^–7^ m^2^ K W^−1^). The *λ* of AlN/PMHS and AlN@PDVB-*co*-PACl/PMHS composites increases with the increase in the mass fraction of AlN. When the mass fraction of AlN@PDVB-*co*-PACl is 85 wt%, AlN@PDVB-*co*-PACl/PMHS composites have the best thermal conductivity, *λ* is 1.89 W m^−1^ K^−1^, which is better than that of *λ* for AlN/PMHS (1.58 W m^−1^ K^−1^). As the filler dosage increases, the overlapping of AlN in the PMHS matrix becomes more pronounced, facilitating the formation of continuous thermal conduction pathways. The thermal conductivity composites with AlN as fillers prepared by researchers over the most years are compared in Table S1. The AlN@PDVB-*co*-PACl/PMHS composites prepared from PDVB-*co*-PACl modified AlN have optimum thermal conductivity.

The effect of PDVB-*co*-PACl grafting on the compatibility of the AlN/PMHS interface is further investigated by testing the binding amount (*β*) of AlN and PMHS on the AlN@PDVB-*co*-PACl surface, and calculating the binding energy (*E*_a_, Eq. S5) of AlN and AlN@PDVB-*co*-PACl to PMHS. The curves plot of the logarithmic versus inverse temperature fit to the amount of PMHS bound to the AlN surface is shown in Fig. 4e, and based on its slope the *E*_a_ of AlN and AlN@PDVB-*co*-PACl with PMHS can be calculated (Table S2). From Table S2, the interfacial binding energy of AlN@PDVB-*co*-PACl/PMHS is 17.13 J mol^−1^, which is higher than that of AlN/PMHS (6.73 J mol^−1^), indicating that AlN@PDVB-*co*-PACl/PMHS has relatively better interfacial compatibility. Because PDVB-*co*-PACl is a branched structure, it contains a large number of reactive vinyl groups at the end of the main chains and branched chains, which form bonds with PMHS matrix through silica-hydrogen addition reaction. Moreover, the PDVB-*co*-PACl contains a large number of branched chains, which are easier to diffuse and tangle with PMHS matrix, resulting in AlN@PDVB-*co*-PACl/PMHS better interface compatibility and greater interface binding energy. AlN@PDVB-*co*-PACl/PMHS composites not only incorporate the high thermal conductivity of AlN itself but also realize further performance enhancement through the modification of PDVB-*co*-PACl. With the increasing demand for efficient thermal management in modern electronic devices, new energy vehicles and aerospace industries, AlN@PDVB-*co*-PACl/PMHS composites will undoubtedly play an increasingly important role in the field of thermal interface materials and lead the new development of thermal management technology in the future. Figure 4f shows the viscosity versus shear rate curves for AlN@PDVB-*co*-PACl composites with different mass fraction. At the same shear rate, the viscosity of AlN@PDVB-*co*-PACl/PMHS composites increases with the increase in the mass fraction of fillers. Because PMHS molecular chains are flexible, intermolecular forces are small, and relative slip is easy [54]. In contrast, AlN is an irregular rigid solid, and the relative slip between AlN and PMHS molecular chains as well as AlN and AlN is difficult, which is not favorable for the flow of the composite system [55]. Therefore, the viscosity of AlN@PDVB-*co*-PACl/PMHS gradually increased with the increase in the mass fraction of fillers. When the mass fraction of AlN@PDVB-*co*-PACl is high (85 and 75 wt%), the viscosity of AlN@PDVB-*co*-PACl/PMHS composites show obvious shear thinning phenomenon [56]. The reason is that the molecular chains in the AlN@PDVB-*co*-PACl/PMHS are highly entangled with each other and the intermolecular forces are high when the system is not subjected to shear or at a low shear rate. And the molecular chains are in the dynamic equilibrium of entanglement and disentanglement, which is macroscopically manifested by the higher viscosity of the system. At high shear rate, the polymer system moves toward unentanglement due to the action of high shear force, and the degree of entanglement is small. Therefore, the intermolecular force decreases, the intermolecular slip is easy, and the macroscopic system viscosity decreases.

### Mechanical and Thermal Properties of AlN@PDVB-co-PACl/PMHS Composites

**Fig. 5:**
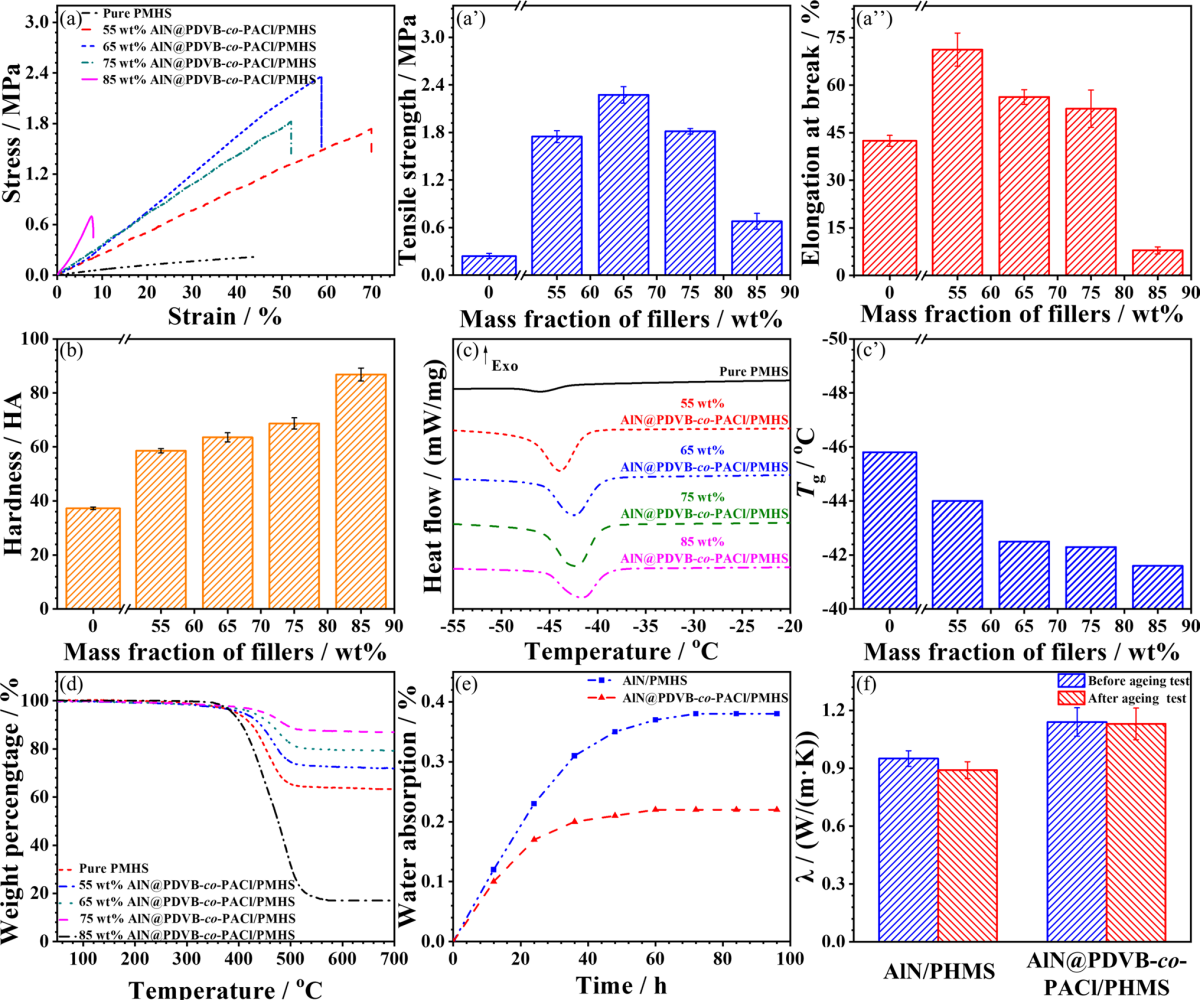
**a** Stress–strain curves, **a’** tensile strength, **a’’** elongation at break and **b** hardness of AlN@PDVB-*co*-PACl/PMHS composites. **c** DSC curves, **c’** *T*_g_ and **d** TGA curves of AlN@PDVB-*co*-PACl/PMHS composites. **e** Water absorption and **f** *λ* of AlN/PMHS and AlN@PDVB-*co*-PACl/PMHS composites before and after the aging test

Figure 5a shows the stress–strain curves of AlN@PDVB-*co*-PACl/PMHS composites. The stress of PMHS and AlN@PDVB-*co*-PACl/PMHS composites under tension increases linearly with the increasing strain until the PMHS and AlN@PDVB-*co*-PACl/PMHS composites break. The stress decreases rapidly after reaching the maximum value without any obvious yielding process. Figure 5a**’–a’’** shows the tensile strength and elongation at break of AlN@PDVB-*co*-PACl/PMHS composites at different mass fraction of AlN@PDVB-*co*-PACl. Both tensile strength and elongation at break of AlN@PDVB-*co*-PACl/PMHS first increase and then decrease with the increasing amount of fillers. When the mass fraction of AlN@PDVB-*co*-PACl is 85 wt%, the tensile strength of AlN@PDVB-*co*-PACl/PMHS composites is 0.68 MPa, which is higher than that of pure PMHS (0.24 MPa), and the elongation at break is 7.9%, which is much lower than the elongation at break of pure PMHS (42%). When the mass fraction of AlN@PDVB-*co*-PACl is 75 wt%, the elongation at break and tensile strength of AlN@PDVB-*co*-PACl/PMHS are 53% and 1.81 MPa, respectively, which are both higher than that of pure PMHS (42% and 0.24 MPa). Both PDVB-*co*-PACl main chains and branched chains of end vinyl groups on the AlN@PDVB-*co*-PACl surface can participate in the curing of PMHS matrix, which enhances the mechanical properties of AlN@PDVB-*co*-PACl/PMHS composites. However, when the mass fraction of AlN@PDVB-*co*-PACl is too high (85 wt%), a large number of defects in the PMHS matrix, which significantly reduces its tensile strength and elongation at break.

Figure 5b shows the hardness of AlN@PDVB-*co*-PACl/PMHS with different mass fraction of AlN@PDVB-*co*-PACl. As exhibited, the hardness improves with the increase in fillers. When the mass fraction of AlN@PDVB-*co*-PACl reaches 85 wt%, the hardness of AlN@PDVB-*co*-PACl/PMHS increased to the maximum of 87 HA from that of the pure PMHS matrix (36 HA). This is because AlN is a diamond-like structure, its deformation under pressure is very small [57], reducing the degree of deformation of AlN@PDVB-*co*-PACl/PMHS composites under pressure and revealing a greater hardness. Meanwhile, functional groups on the AlN@PDVB-*co*-PACl surface can participate in the curing of the PMHS matrix, which increases the density of the PMHS crosslinked network [58], resulting in increased hardness of the AlN@PDVB-*co*-PACl/PMHS composites. Figure 5c–c’ shows the DSC curves and corresponding glass transition temperatures (*T*_g_) of the AlN@PDVB-*co*-PACl/PMHS composites at different mass fraction of AlN@PDVB-*co*-PACl. The *T*_g_ of AlN@PDVB-*co*-PACl/PMHS is proportional to the amount of fillers. When the mass fraction of AlN@PDVB-*co*-PACl is 85 wt%, the *T*_g_ of AlN@PDVB-*co*-PACl/PMHS is − 41.6 °C, which is 4.2 °C higher than that of pure PMHS matrix. The AlN@PDVB-*co*-PACl surface contains a large number of main chains and branched chains of end vinyl, which can participate in the curing reaction of PMHS matrix, increasing the cross-linking density of AlN@PDVB-*co*-PACl/PMHS composites. Therefore, the movement of PMHS molecular chains is limited, and the *T*_g_ of AlN@PDVB-*co*-PACl/PMHS is increased with the increasing amount of fillers. Figure 5d shows the TGA curves of AlN@PDVB-*co*-PACl/PMHS composites at different mass fraction of AlN@PDVB-*co*-PACl. Pure PMHS and AlN@PDVB-*co*-PACl/PMHS begin to lose weight at around 380 °C, and the mass equilibrates at around 560 °C, which is mainly attributed to thermal cleavage of the PMHS molecular chains at this temperature. In addition, when the mass fraction of AlN@PDVB-*co*-PACl is 55, 65, 75, and 85 wt%, the residual masses of AlN@PDVB-*co*-PACl/PMHS composites are 63.1, 71.6, 79.2, and 86.7 wt%, respectively, which are corresponding to the amount of fillers. The *T*_5_ of AlN@PDVB-*co*-PACl/PMHS (corresponding temperature at a weight loss of 5 wt%, Fig. S5) is proportional to the amount of fillers. When the mass fraction of AlN@PDVB-*co*-PACl is 85 wt%, the *T*_5_ of AlN@PDVB-*co*-PACl/PMHS is 449.2 °C, which is 61.5 °C higher than that of pure PMHS matrix (387.7 °C). Because a large number of main chains and branched chains of end vinyl on the AlN@PDVB-*co*-PACl surface can participate in the curing reaction of PMHS matrix. It helps to increase the cross-linking density of the curing network and improve the heat resistance of AlN@PDVB-*co*-PACl/PMHS composites.

Figure 5e illustrates the water absorption of AlN/PMHS and AlN@PDVB-*co*-PACl/PMHS composites when the mass fraction of AlN or AlN@PDVB-*co*-PACl is 75 wt%. The water absorption of AlN/PMHS and AlN@PDVB-*co*-PACl/PMHS composites increase with the extension of time, and reach saturation after 80 h. Because the rapid diffusion of water from the composites surface to the interface of AlN/PMHS or AlN@PDVB-*co*-PACl/PMHS increases the water absorption. When the water absorption at the internal interface of the composites tends to be saturated, the water absorption rate is gradually stable. After impregnating at room temperature for 80 h, the water absorption of AlN@PDVB-*co*-PACl/PMHS is 0.22%, which is significantly lower than that of AlN/PMHS (0.38%). Compared with AlN/PMHS, AlN@PDVB-*co*-PACl/PMHS composites have relatively better interfacial compatibility, with relatively fewer defects and pores at the interface, which is more conducive to hindering the entry of water molecules. In addition, PDVB-*co*-PACl results in a higher molecular chain network density at the interface of AlN@PDVB-*co*-PACl/PMHS composites, which makes it more difficult for water molecules to diffuse. This in turn provides the AlN@PDVB-*co*-PACl/PMHS composites with relatively lower water absorption and relatively better water resistance. Figure 5f shows the *λ* before and after the aging test of AlN/PMHS and AlN@PDVB-*co*-PACl/PMHS composites (80 h in 90 °C deionized water) when the mass fraction of AlN or AlN@PDVB-*co*-PACl is 75 wt%. After the aging test, the *λ* of AlN/PMHS composites decreases from 0.95 W to 0.89 W m^−1^ K^−1^. In contrast, the *λ* of AlN@PDVB-*co*-PACl/PMHS composites retain relatively constant of 1.13 compared to 1.14 W m^−1^ K^−1^ about retaining 99.1%, whereas the *λ* of the blended AlN/PMHS composites decreases sharply to 93.7%. Because the main chains and branched chains on the AlN@PDVB-*co*-PACl surface contain a large amount of reactive vinyl, which participate in PMHS matrix curing. Results in a higher molecular chain network density at the interface of AlN@PDVB-*co*-PACl/PMHS, and the more stable interface under hot and humid conditions. Therefore, the AlN@PDVB-*co*-PACl/PMHS composites have better aging resistance and more stable thermal conductivity. At the same time, the excellent aging resistance properties of AlN@PDVB-*co*-PACl/PMHS composites ensure the stability of the material in the long-term use of the process, extending the life of electronic equipment.

## Conclusions

The copolymer of PDVB-*co*-PACl is synthesized by ATRP using *t*BA and DVB as the monomer. PDVB-*co*-PACl is utilized to graft on the surface of spherical AlN to prepare AlN@PDVB-*co*-PACl. PMHS is then used as the matrix to prepare thermally conductive AlN@PDVB-*co*-PACl/PMHS composites through blending and curing. The grafting of PDVB-*co*-PACl synchronously enhances the hydrolysis resistance of AlN and its interfacial compatibility with PMHS matrix. When the molecular weight of PDVB-*co*-PACl is 5100 g mol^−1^ and the grafting density is 0.8 wt%, the composites containing 75 wt% of AlN@PDVB-*co*-PACl exhibit the optimal comprehensive performance. The *λ* of the composites is 1.14 W m^−1^ K^−1^, which enhances by 20% and 420% compared to the *λ* of simply physically blended AlN/PMHS composites and pure PMHS, respectively. Meanwhile, AlN@PDVB-*co*-PACl/PMHS composites display remarkable hydrothermal aging resistance by retaining 99.1% of its *λ* after soaking in 90 °C deionized water for 80 h, whereas the *λ* of the blended AlN/PMHS composites decreases sharply to 93.7%.

## Supplementary Information

Below is the link to the electronic supplementary material.

Supplementary file1 (DOCX 2770 KB)
