# Supplementary material for: Functionalized Aluminum Nitride for Improving Hydrolysis Resistances of Highly Thermally Conductive Polysiloxane Composites
Source: Nanomicro Lett. 2025 Feb 6;17:134. doi: 10.1007/s40820-025-01669-5 (PMC11799469; doi:10.1007/s40820-025-01669-5)
Supplement: Supplementary file 1 — Supplementary file1 (DOCX 2770 KB) [file 40820_2025_1669_MOESM1_ESM.docx]

Supporting Information for

**Functionalized Aluminum Nitride for Improving Hydrolysis Resistances of Highly Thermally Conductive Polysiloxane Composites**

Mukun He^1#^, Lei Zhang^1#^, Kunpeng Ruan^1^, Junliang Zhang^1^, Haitian Zhang^1^, Peng Lv^3^, Yongqiang Guo^1^, Xuetao Shi^1^, Hua Guo^1^*, Jie Kong^1^, Junwei Gu^1, 2^*

^1^Shaanxi Key Laboratory of Macromolecular Science and Technology, School of Chemistry and Chemical Engineering, Northwestern Polytechnical University, Xi’an, Shaanxi 710072, P. R. China

^2^Chongqing Innovation Center, Northwestern Polytechnical University, Chongqing 401135, P. R. China

^3^School of Materials Science and Engineering, Shandong University of Technology, Zibo 255000, P. R. China

# Mukun He and Lei Zhang contributed equally to this work.

*Corresponding authors. E-mail: [gh@nwpu.edu.cn](mailto:gh@nwpu.edu.cn) (Hua Guo); gjw@nwpu.edu.cn & [nwpugjw@163.com](mailto:nwpugjw@163.com) (Junwei Gu)

**S1 Main Materials**

Polymethylhydrosiloxane (PMHS, Industrial grade) was received from Siquan New Materials Co., Ltd. (Guangdong, China). Aluminum nitride (AlN), divinyl benzene (DVB, Analytical grade), cuprous bromide (CuBr, Analytical grade), and sulfoxide chloride (SOCl_2_, Analytical grade) were all supplied by Shanghai Macklin Biochemical Technology Co., Ltd (Shanghai, China). Tert-butyl acrylate (*t*BA, Analytical grade) was purchased from Jiuding Chemical Technology Co. Ltd. (Shanghai, China). Pentamethyldivinyltriamine (PMDETA) was obtained from Guangdong Guanghua Technology Co., Ltd. (Guangdong, China). Methyl 2-bromopropionate (CH_3_CHBrCOOCH_3_) and N,N-Dimethylacetamide (DMAc) were both obtained from Sinopharm Chemical reagent Co., Ltd (Shanghai, China).

**S2 Characterizations**

^1^H nuclear magnetic resonance (^1^H NMR) spectra were recorded on Bruker AV 400 NMR spectrometer (Bruker, Co., Germany) with tetramethylsilane as internal standard and deuterated chloroform as the solvent. Fourier transform infrared (FT-IR) spectra were captured on Bruker Tensor 27 equipment (Bruker Co., Germany) by attenuated total reflection module. The molecular weight and molecular weight distribution of the polymer were determined by size exclusion chromatography (SEC) with tetrahydrofuran (THF) as solvent and flow rate fixed at 1.0 mL/min. X-ray photoelectron spectroscopy (XPS-Axis Ultra DLD, Kratos Co., UK) was used to analyze the elemental composition and binding energy variations. X-ray diffraction (XRD) curves were obtained using the X-ray diffractometer (XRD-6100, Shimadzu Co., Japan) with scanning speed of 5^o^/min, diffraction angle 2θ=10^o^~80^o^ and 0.02^o^/step. Thermal gravimetric analyses (TGA) were carried out by STA 449F3 (NETZSCH Co., Germany) with heating rate of 10^o^C/min under argon atmosphere. Differential scanning calorimetry (DSC) curves of the samples were conducted under nitrogen atmosphere with heating rate of 10^o^C/min by DSC1 (Mettler-Toledo Co., Switzerland). Transmission electron microscopy (TEM) images were obtained using the Talos F200X/TEM microscope (FEI Corp., America). Thermal conductivity coefficients were measured using TPS2200 Hot Disk thermal constant analyzer (AB Corp., Sweden), in accordance with the standard ISO 22007-2:2015. Mechanical properties were evaluated using electronic universal testing machine (Sitai Instrument Co., China) in accordance with GB/T 528-2009 at a rate of 20 mm/min. The hardness of the samples was obtained by a shore A hardness tester of ALX-AC (Aolong Co., China).

According to the Hamilton-Hasselman model [S1], the interfacial thermal resistance (*ITR*) of the composites can be calculated by Eqs. S1-S4.

$\frac{\lambda_{c}}{\lambda_{m}}=\frac{\lambda_{f}\left[ 1+\left( n-1 \right)\alpha\right]+{\left( n-1 \right)\lambda}_{m}+\left( n-1 \right)V_{f}\left[ \lambda_{f}\left( 1-\alpha\right)-\lambda_{m} \right]}{\lambda_{f}\left[ 1+\left( n-1 \right)\alpha\right]+\left( n-1 \right)\lambda_{m}-V_{f}\left[ \lambda_{f}\left( 1-\alpha\right)-\lambda_{m} \right]}$ （S1）

$\alpha=\frac{\alpha_{k}}{a}$ （S2）

$\alpha_{k}=(ITR)\lambda_{m}$ （S3）

$n=\frac{3}{\Psi}$ （S4）

*λ_c_* is the thermal conductivity of the composites. *λ*_m_ is the thermal conductivity of the matrix. *λ_f_* is the thermal conductivity of the thermal conductive fillers. *α* is the phonon transmission probability. *a* is the average particle size of the thermal conductive fillers. *V_f_* is the volume fraction of the thermal conductive fillers. *Ψ* is the sphericity of the particles, which is usually taken as 0.7.

The solvent extraction method was used to test the surface bound PMHS of AlN and AlN@PDVB-*co*-PACl, which can be used to calculate the interfacial binding energies (*E*_a_) of AlN and AlN@PDVB-*co*-PACl with PMHS. The uncured blends of AlN and AlN@PDVB-*co*-PACl with PMHS were submerged in solvents of different temperatures and the solvents were changed every 10 h. The residual PMHS on the surface of the filler was measured after 72 h as *β*, which was plotted against temperature as shown in Equation S5:

$ln\beta=lnA-E_{a}/RT$ （S5）

*β* is the binding amount of PMHS on the surface of AlN and AlN@PDVB-*co*-PACl. *E*_a_ and *A* are the interfacial binding energy and the finger-forward factor for the interaction of AlN and AlN@PDVB-*co*-PACl with PMHS, respectively. *R* is a constant of 8.314 J/(mol·K). *T* is the thermodynamic temperature.

**S3 Supplementary Figures**

**Fig. S1** SEC curves of PDVB-*co*-PACl


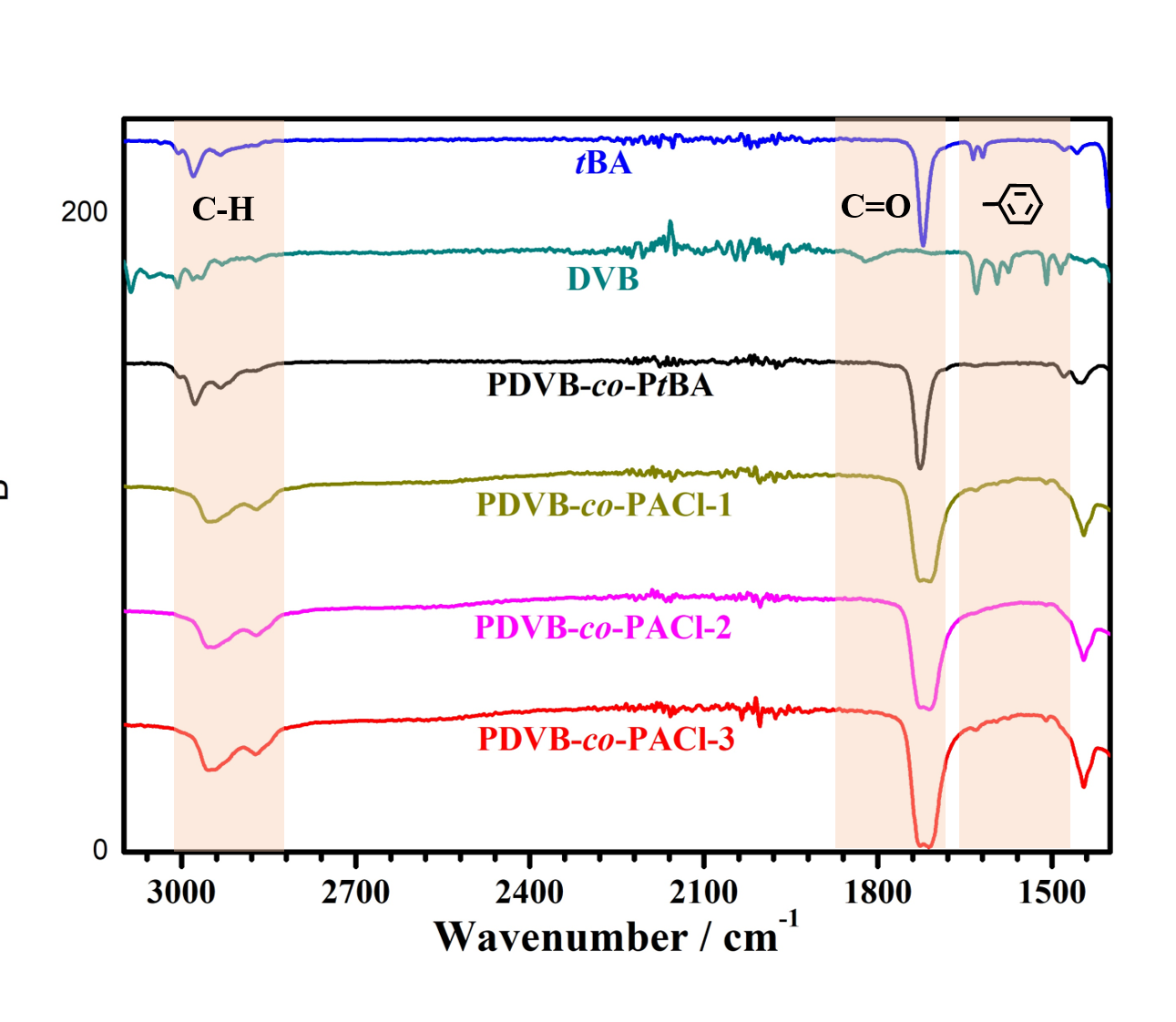


**Fig. S2** FT-IR spectra of *t*BA, DVB, PDVB-*co*-P*t*BA, and PDVB-*co*-PACl


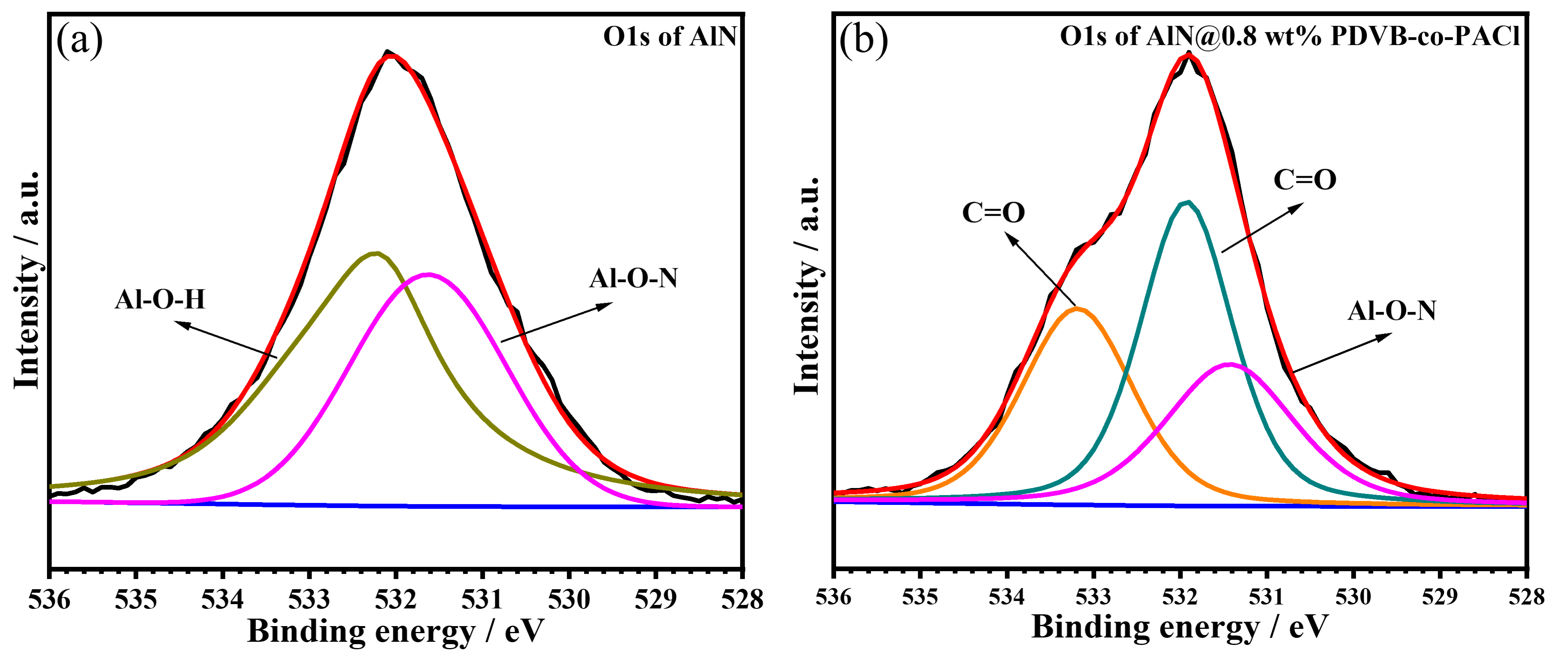


**Fig. S3** **a** O 1s spectra of AlN and **b** AlN@PDVB-*co*-PACl


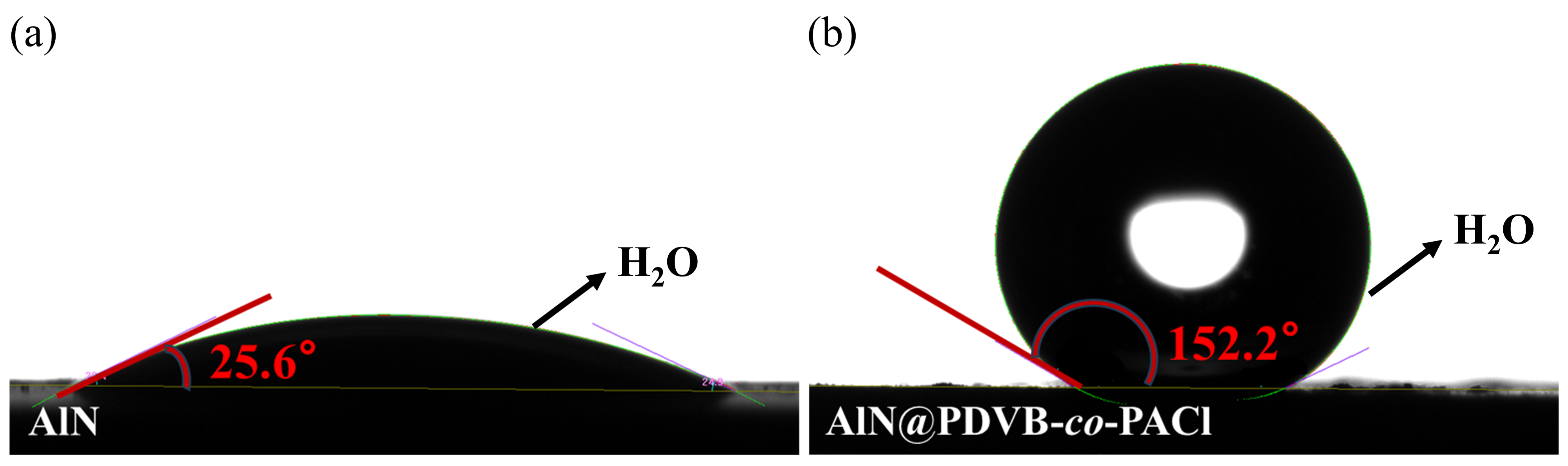


**Fig. S4** Water contact angles of **a** AlN and **b** AlN@PDVB-*co*-PACl


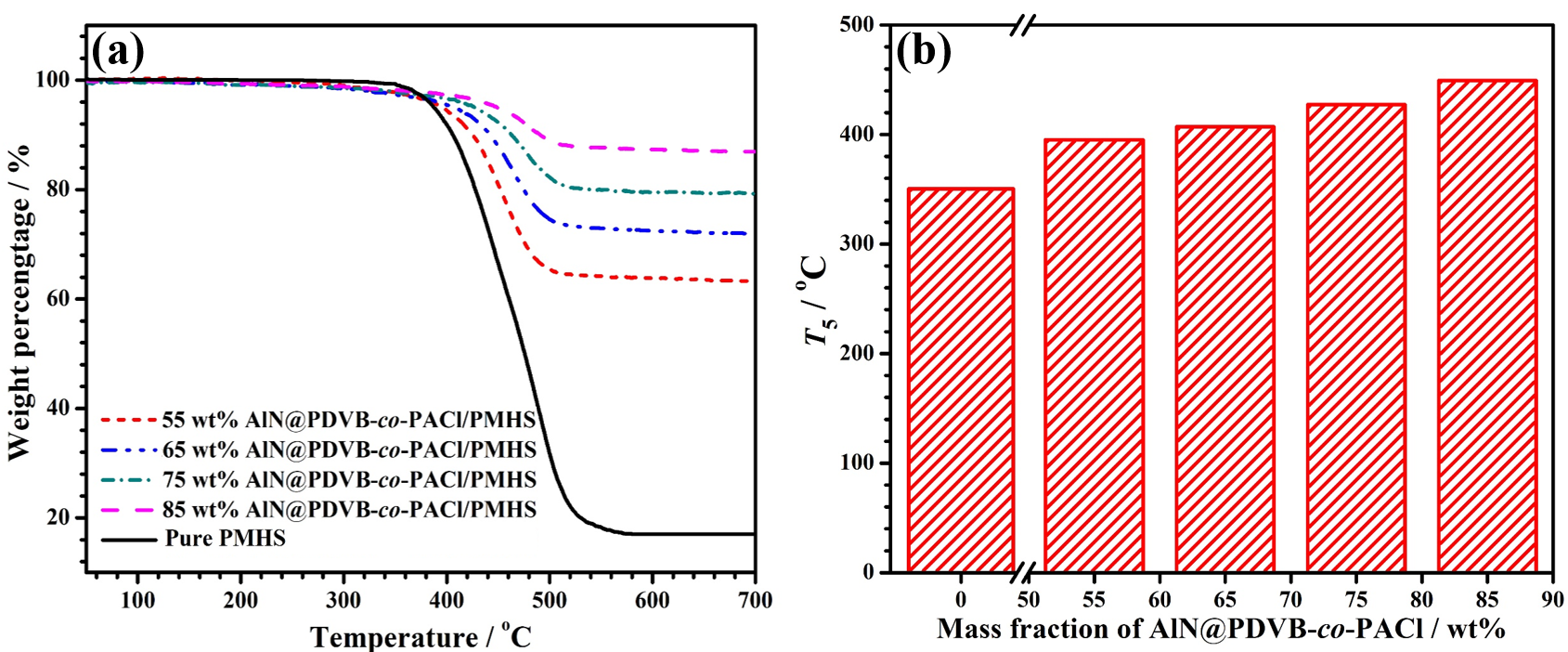


**Fig. S5** *T*_5_ of AlN@PDVB-*co*-PACl/PMHS thermally conductive composites

**S4 Supplementary Table**

**Table S1** Thermal conductivity of thermally conductive composites prepared with AlN as fillers

| **Sample** | **Dosage** | ***λ* / W/(m·K)** | **Refs.** |
| --- | --- | --- | --- |
| AlN/EP | 40 wt% | 0.70 | [S2] |
| AlN/KH560/EP | 40 wt% | 0.78 | [S2] |
| micro-nano AlN/PTFE | 30 vol% | 0.84 | [S3] |
| AlN/PI | 9 vol% | 0.65 | [S4] |
| AlN/KH560/PI | 9 vol% | 0.68 | [S4] |
| AlN-VTMS/PMHS | 60 wt% | 0.31 | [S5] |
| AlN/PU | 50 wt% | 0.48 | [S6] |
| AlN@(PEDOT:PSS)/PU | 50 wt% | 0.76 | [S6] |
| AlN-PPy-KH570/SR | 50 wt% | 0.37 | [S7] |
| ***AlN/PMHS*** | ***75 wt%*** | ***0.95*** | ***This work*** |
| ***AlN@PDVB-co-PACl/PMHS*** | ***75 wt%*** | ***1.14*** | ***This work*** |

**Table S2** *E*_a_ values of AlN/PMHS and AlN@PDVB-*co*-PACl/PMHS

| **Samples** | ***Slope*** | ***E_a_* (J/mol)** |
| --- | --- | --- |
| **AlN/PMHS** | 0.81 | 6.73 |
| **AlN@PDVB-*co*-PACl/PMHS** | 2.06 | 17.13 |

**Supplementary References**

[S1] K. Ruan, X. Shi, Y. Guo, J. Gu, Interfacial thermal resistance in thermally conductive polymer composites: A review. Compos. Commun. **22**, 100518 (2020). <https://doi.org/10.1016/j.coco.2020.100518>

[S2] A.-j. Ma, W. Chen, Y. Hou, Enhanced Thermal Conductivity of Epoxy Composites with MWCNTs/AlN Hybrid Filler. Polym-Plast. Technol. **51**, 1578-1582 (2012). <https://doi.org/10.1080/03602559.2012.716479>

[S3] C. Pan, K. Kou, Q. Jia, Y. Zhang, Y. Wang et al., Fabrication and characterization of micro-nano AlN co-filled PTFE composites with enhanced thermal conductivity: a morphology-promoted synergistic effect. J. Mater. Sci-Mater. El. **27**, 11909-11916 (2016). <https://doi.org/10.1007/s10854-016-5336-1>

[S4] L. Liu, C. Cao, X. Ma, X. Zhang, T. Lv, Thermal conductivity of polyimide/AlN and polyimide/(AlN+BN) composite films prepared by in-situ polymerization. J. Macromol. Sci. A **57**, 398-407 (2020). <https://doi.org/10.1080/10601325.2019.1703555>

[S5] J. Zheng, S. He, J. Wang, W. Fang, Y. Xue et al., Performance of Silicone Rubber Composites Filled with Aluminum Nitride and Alumina Tri-Hydrate. Materials **13**, 2489 (2020). <https://doi.org/10.3390/ma13112489>

[S6] C.-T. Yang, H.-I. Hsiang, T.-S. Huang, P.-C. Huang, Y.-K. Han, Thermal conductivity and dielectric properties of PEDOT:PSS-AlN filler reinforced water-soluble polymer composites. Ceram. Int. **43**, S710-S716 (2017). <https://doi.org/10.1016/j.ceramint.2017.05.271>

[S7] K. Yang, Y. Chen, H. Dong, J. Jiao, X. Lang et al., Synchronously enhanced thermal conductivity and dielectric properties of silicone rubber composites filled with the AlN-PPy-KH570 multilayer core-shell hybrid structure. Polym. Eng. Sci. **64**, 5842-5853 (2024). <https://doi.org/10.1002/pen.26956>
